# Direct adsorption sampling and ambient mass spectrometry analysis of tobacco smoke with porous paper strips

**DOI:** 10.3389/fchem.2022.1037542

**Published:** 2022-10-26

**Authors:** Ji Yang, Wen Xiong, Chunbo Liu, Juan Li, Ruizhi Zhu, Jianjun Xia, Zhijiang Yin, Ran Tian, Shiyun Tang, Zhenjie Li, Hui Li, Ying Han, Xiaoxi Si, Wei Jiang, Pei He, Fengmei Zhang, Yanqin Xu, Zhihua Liu

**Affiliations:** Technology Center, China Tobacco Yunan Industrial Co., Ltd., Kunming, China

**Keywords:** paper spray, tobacco smoke, ambient mass spectrometry, paper strip, sampling

## Abstract

Chemical analysis of atmospheric aerosols by conventional analytical methods is usually required to perform complicated and time-consuming sample preparation processes. In recent decades, ambient ionization mass spectrometry (AI-MS) methods have been proven to be simple, rapid, and effective analytical tools for direct analysis of various complex samples. In this work, we applied porous paper filters for direct adsorptive sampling of tobacco smoke, and then the sampled paper filters were performed the emitters of the paper spray ionization (PSI) device. An auto-sampling device was made to control the generation and collection of tobacco smoke. Nicotine, the typical compound of tobacco smoke, was used to optimize the key conditions of auto-sampling. Moreover, different types of tobacco smoke were also compared with multivariate variable analysis, and the makers of tobacco smoke from different sources of tobacco smoke were investigated. By using this method, direct sampling and analysis of a single tobacco sample can be completed within minutes. Overall, our results show that PSI-MS is a powerful tool that integrates collection, extraction, ionization, and identification analytes in smoke.

## Introduction

Environmental tobacco smoke is one kind of aerosols produced by the incomplete combustion of tobacco during the smoking of cigarettes and other tobacco products (Kavouras et al., 1998; Brauer et al., 2000; Zhu et al., 2022). Tobacco smoke is one of the air pollutants that contain many harmful chemicals to human health (Mueller et al., 2011). It is reported that thousands of chemicals can be found in tobacco smoke, many of those are known to be harmful; human breathing even small amounts of tobacco smoke can be harmful (Kitamura and Kasai, 2007; Rogers, 2008; Fagerström, 2011). It is well-known that tobacco smoking could cause many diseases, including cancer, heart disease, stroke, diabetes, and other chronic diseases (Eyre et al., 2004; Halpin et al., 2010; Stanton et al., 2016). Researchers should face up to the fact that although the law tightens anti-smoking restrictions in many countries, smoking is still allowed in some special smoking areas (Louka et al., 2006). Gaining insight into tobacco smoke would be useful for understanding the properties of smoking behavior. Chemical analysis is one of the powerful analytical strategies for understanding tobacco smoke at the molecular level (Borgerding and Klus, 2005; Orr, 2014; Jiang, 2020).

There are many established analytical methods for chemical analysis of tobacco smoke. Investigating the chemical composition of tobacco smoke has been successfully performed by gas chromatography (GC), liquid chromatography (LC), and inductively coupled plasma (ICP) coupled with electrochemical, fluorescence, and mass spectrometry (MS) (Shuker and Bartsch, 1994; Zhang et al., 2011; Musharraf et al., 2012; Cuello-Nuñez et al., 2018; Haiduc et al., 2020). Among these methods, MS-based approaches can measure the chemical compositions of environmental samples. To date, various MS methods have been developed as reference techniques for comprehensive analysis of complex samples (Lee et al., 2008; Várhegyi et al., 2009; Hossain and Salehuddin, 2013; Hu and Ouyang, 2021; Yuan and Hu, 2021; Hu, 2022). Moreover, MS-based methods have also been applied for monitoring air pollutants such as tobacco smoke (Bernert Jr et al., 2000). Conventional MS-based methods for analyzing air pollutants require sample preparation processes. Therefore, reducing or removing the sample preparation and chromatographic separation steps before MS analysis is highly demanded in the investigation of tobacco smoke. For example, pre-collection and sample pretreatment of tobacco smoke for subsequent analysis using different MS-based approaches have been successfully performed in previous studies (Lee et al., 2008; Várhegyi et al., 2009; Hossain and Salehuddin, 2013). However, there is a limitation as these chromatography-based methods are time-consuming and labor-intensive processes, which are not amenable to rapid analysis and evaluation of tobacco smoke. Therefore, there is high demand for developing new MS-based methods for rapid evaluation of tobacco smoke.

Ambient MS methods are of great interest for direct analysis of complex samples, because raw complex samples can be directly ionized without sample pretreatment process and chromatography separation. Ambient MS is pioneered by the development of desorption electrospray ionization (DESI), which allows direct desorption/ionization of raw samples in different forms (Cooks et al., 2006). To date, various ambient ionization methods have been developed for ambient MS analysis of raw samples (Feider et al., 2019; Tata et al., 2022). Remarkable ambient MS methods were further developed with ESI on different substrates. Electrospray ionization (ESI) is one of the most powerful ionization techniques for MS-based qualitative and quantitative detection of analytes from complex samples. The raw complex samples were usually prepared into a liquid solution with water, methanol, or other organic solvents, and were then introduced to ESI source *via* capillary with the assistance of high voltage and sheath gas. With the emerging development of ambient ionization for MS analysis, various new ESI-related ambient ionization techniques have been introduced for rapid analysis of complex samples in different fields including environmental analysis. In the late 1990s, a non-capillary ESI method was introduced in which the capillary was replaced by a copper wire for direct ionization of liquid samples (Hong et al., 1999). Under such noncapillary ESI, the raw sample can be directly loaded on a solid substrate for direct ESI of raw samples (Yang et al., 2013). Recently, remarkable achievements of ambient ESI have also been made by different solid substrates, various materials, such as paper strips (Yao et al., 2020; Cai et al., 2021), wooden tips (Hu and Yao, 2022; Millán-Santiago et al., 2022), metal needles (Mandal et al., 2011; Saha et al., 2013), sharp blades (Gómez-Ríos and Pawliszyn, 2014), medical swabs (Pirro et al., 2015; Wu et al., 2020), fibers (Filho et al., 2020; Wu et al., 2021), foils (Hu et al., 2014), biological tissues (Hu et al., 2012), living organisms (Hu et al., 2013; Li et al., 2021), and others (Klampfl and Himmelsbach, 2015) can be modified to be a tiny tip for loading sample and generating spray ionization.

Among these solid materials, paper strip has the unique features for sampling and generating spray ionization. Ambient ESI on paper strip, also called paper spray ionization (PSI), has been proved a powerful analytical tool for direct sample analysis (Liu et al., 2010). Undoubtedly, paper strip can be used for direct sampling by loading or touching raw complex samples (Deng and Yang, 2013; Deng et al., 2016; Yang et al., 2018; Nguyen et al., 2022). Liquid, semi-liquid, powders, and biological samples can also be directly analyzed by using PSI-MS. In PSI-MS, soft, disposable, and low-cost paper material is quite convenient for direct sampling of raw samples. In general, under PSI-MS processes, the compounds of interest are extracted from the paper strip by loading a drop of organic solvent, and then generated spray ionization for MS analysis by using a strong electric field that applies a high voltage on paper strip. Particularly, without any sample preparation and chromatographic separation, PSI-MS can be performed for the identification of target analytes with good analytical performances. It is reported that many biological and environmental samples can be analyzed by PSI-MS (McBride et al., 2019). Therefore, paper strip is expected to characterize tobacco smoke.

To the best of our knowledge, PSI-MS has rarely been used for direct analysis of tobacco smoke. Herein, we aim to make a new attempt at the direct collection and direct analysis of tobacco smoke. In this work, tobacco smoke was collected onto porous paper using a homemade auto-sampling device, and the sampled paper filters were then performed PSI-MS analysis. The sampling conditions were optimized. Different types of tobacco smoke were also investigated with multivariate variable analysis. Our results show that paper sampling coupled with PSI is a promising tool for smoke collection and identification.

## Materials and methods

### Chemicals and materials

Nicotine standard solutions were purchased from Cerilliant (Round Rock, TX, United States). Pure methanol was purchased from Sigma (St. Louis, United States). Cigarettes were purchased from a local supermarket. Filter papers and chromatography papers were purchased from different suppliers. Type 1 of filter paper was purchased from Home Administration Filter Paper Factory (Fushun, China); type 2 of filter paper was purchased from General Electric Biotechnology Co., Ltd., Hangzhou, China; type 3 of chromatography paper was purchased from Beyotime Biotechnology Co., Ltd., Shanghai, China; type 4 of chromatography papers were purchased, North Wood Pulp & Paper Co., Ltd., Fuyang, Hangzhou, China; type 5, thick chromatography papers s were purchased from Cambridge Filter Service Co. (Mesa, AZ, United States).

### Paper sampling of tobacco smoke

**FIGURE 1:**
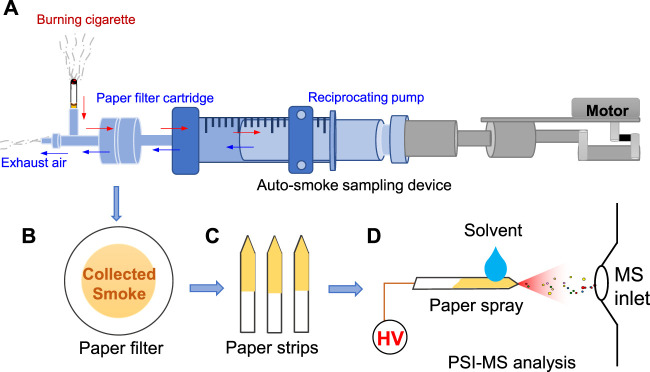
Paper sampling and PSI-MS analysis of tobacco smoke: **(A)** sampling device, **(B)** paper filter after sampling, **(C)** paper strips, **(D)** PSI-MS method.

As shown in Figure 1A, circular paper filter (diameter: 44 mm) was mounted on the auto-smoking glass device for the collection of tobacco smoke. The suction volume and frequency can be controlled by the moto of auto-smoking device. Typically, the suction volume is simulated to human smoking at ∼ 35 ml for a puff of smoke. In this work, each cyclic auto-sampling suction is defined by extracting air of 35 ml and then exhausting air of 35 ml in this work by a reciprocation pump. After sampling, paper filter (Figure 1B)was removed from the auto-smoking device, and then was cut into a small strip (22 × 3 mm) with a triangle tip-end (< 0.1 mm) to prepare for subsequent PSI-MS analysis (Figure 1C). In the initial experiments, the cumulative suction was set 3 times, while the suction times was set at 24 times per minutes. There are same conditions of cumulative suction are suction times for comparing each type of paper strip.

### Paper spray ionization-mass spectrometry analysis

To perform PSI-MS analysis, the paper strip was mounted on a commercial nanoESI device (Thermo Fisher Scientific, Bremen, Germany) followed previous studies (Liu et al., 2010; Cai et al., 2021), as shown in Figure 1D. By placing nanoESI capillary, paper strip was placed in the front of the mass spectrometer at distances of 1.0 cm horizontal from the paper tip-end to the MS inlet. Pure methanol (i.e., 2.0 μl) which is commonly used in spray ionization in ambient MS plays a significant role in PSI (Aliaga-Aguilar, 2018; Cao and Huang, 2018; McBride et al., 2019), was directly loaded onto a paper tip to extract analytes and generate spray ionization. By applying a high voltage (2.5 kV) onto paper tip, spray ionization could be generated from paper tip-end to acquire a characteristic mass spectrum. The capillary temperature was set at 150°C. The high voltage was supplied from the Orbitrap-LTQ mass spectrometer (Thermo Fisher Scientific, Bremen, Germany) to paper tip. To conduct the tandem MS experiments, precursor ions were selected with an isolation window at 0.4 Da. Each sample was analyzed at least three times to obtain reliable data. Morphologies of all paper strips were examined by a scanning electron microscope (SEM, SU8010, Hitachi, Japan).

### Data analysis

Mass spectral data acquisition and instrumental control were conducted by using Xcalibur 3.0 software (Thermo Fisher Scientific, Bremen, Germany). The acquisition speed was 6 scans/sec, Typically, data from the first 1 min were averaged to generate the mass spectra. All the mass spectra were directly obtained. Principal component analysis (PCA) and partial least squares discriminant analysis (PLS-DA) were carried out using Simca software (Umetrics, Sweden) as described previously (Li et al., 2019). For each MS data, the normalized intensities of those monoisotopic peaks at the mass range from m/z 100 to 500 with signal intensities higher than 0.1% were input to the Simca software for the PCA and PLS-DA analysis. The VIP value of interest ions that are larger than 1.0 were considered the special markers in this work.

## Results and discussion

### Direct collection and analysis of tobacco smoke

**FIGURE 2:**
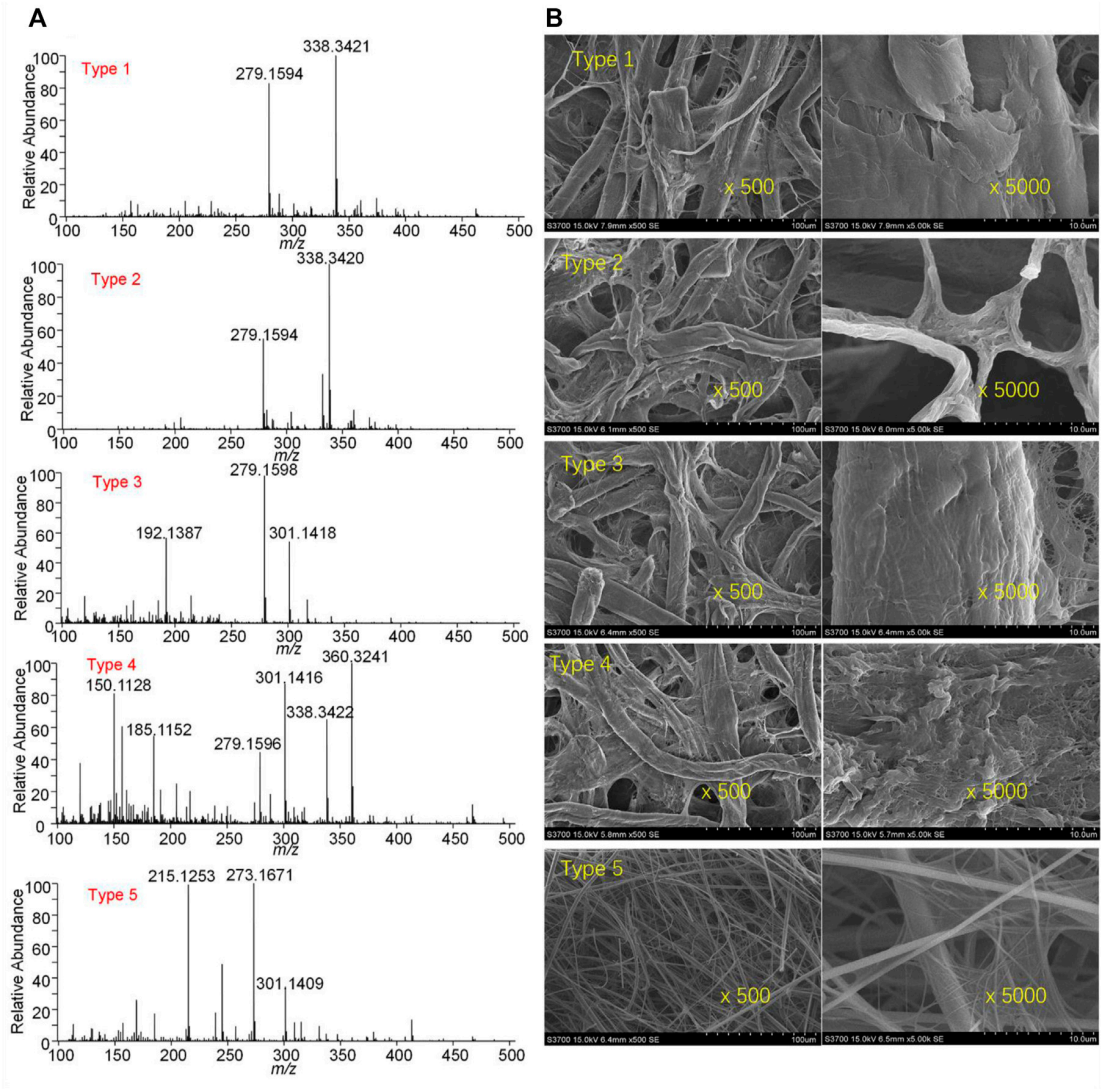
Characterization of different types of paper strips: **(A)** chemical analysis of blank paper strips, **(B)** SEM images of paper strips.

Figure 2A shows blank PSI mass spectra obtained by adding pure methanol onto different types of paper strips. Many peaks were observed from the different types of paper strips. Particularly, it is found that some typical plasticizers such as dibutyl phthalate at m/z 279.1594 [M + H]^+^ and at m/z 301.1418 [M + Na]^+^. These chemicals are possibly from the chemical residues in the paper processing and making industry. The chemical characterization of different types using PSI indicates that there are many matrices in paper surface and pores. Moreover, morphologies of all paper strips were further examined by SEM, as shown in Figure 2B. It is found that different size of pore can be seen in the paper strip. Particularly, there is the smallest pore in the type-3, while smallest fibers were found in type-5. In these fibers, dissolvable chemicals could be resided into fibers and thus be detected in PSI-MS. Therefore, to obtain the clear PSI mass spectra, mass spectra of sample should be subtracted with these blank spectra.

**FIGURE 3:**
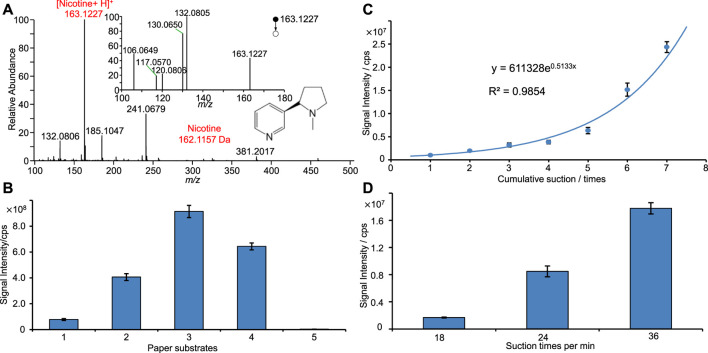
PSI-MS analysis of tobacco smoke: **(A)** typical mass spectrum of tobacco smoke, **(B)** plots of nicotine signal against different paper filters, **(C)** plots of nicotine signal against sampling times, **(D)** plots of nicotine signal against sampling frequencies.

Figure 3A shows the typical PSI-MS spectrum of tobacco smoke. Nicotine (monoisotopic mass: 162.1157 Da), the typical compound of tobacco cigarettes, is clearly observed as protonated ions at *m/z* 163.1227 [M + H]^+^ and was dominated as the base peak in the spectrum. Upon MS/MS experiment, major fragmental ions at *m/z* 132.0805, 130.0650, 120.0806, 117.0570, and 106.0649 were observed (inset of Figure 3A). These fragmental ions are in good agreement with previous work on the identification of nicotine (Yuan et al., 2020). Therefore, Figure 3A clearly shows that the nicotine was identified from tobacco smoke. Figure 3A also displays many other peaks with high signal-to-noise, e.g., m/z 132.0806, 185.1047, 241.0679, which are at low abundance. These peaks could be ascribed to the other special chemicals in tobacco smoke.

To investigate the suction frequency of auto-smoking sampling, different sampling frequencies and paper filters were compared by monitoring the nicotine intensity in this work. Figure 3B shows that nicotine signal was obtained from different sampling conditions, showing that there is the highest signal obtained from the type 3 paper filter, probable due to its compact structure of pore and fibers (Figure 3B). Although all paper substrates are porous, different pores and structures (Figure 3B) could affect the absorptive sampling of chemicals and parties from smoke samples. Figure 3C shows the plot of nicotine intensity against the cumulate times of auto-sampling suction. The nicotine signal is significantly increased with the increasing sampling times. Moreover, an exponential function relation (y = 611328e^0.5133x^, R^2^ = 0.9854) is found from such plot with good coefficient. It is reasonable to find that more nicotine can be sampled onto paper filter under a longer sampling time. As shown in Figure 3D, it is found that the intensity of nicotine was increased with increasing the sampling frequency, probably that high sampling frequency could provide more tobacco smoke onto paper fiber and thus less volatiles were lost in sampling.

Moreover, the analytical performances of PSI-MS for detecting nicotine in tobacco smoke was further investigated. As shown in Table 1. Linearity ranges of nicotine in type-3 paper is 1–500 ng/ml with a acceptable a coefficient (R^2^ = 0.98). The limit of detection (LOD) and limit of quantification (LOQ) was found to be 0.2 ng/ml and 1.0 ng/ml, respectively, which are comparable traditional methods such as GC-MS and LC-MS (Table 1). These results showed that PSI-MS is a sensitive method for nicotine detection. The reproducibility was evaluated by determining nicotine at 100 ng/ml in methanol, showing the relative standard deviation (RSD) of six repeated measures is 13.5%, which is larger than traditional methods for detecting nicotine in different samples (Table 1). The precision of nicotine (100 ng/ml in methanol) was found to be 76–124%, which are also slightly larger than the results obtained by traditional methods. Because PSI-MS is one of ambient MS techniques, there are usually some acceptable fluctuations in experiments. However, compared to traditional methods, PSI-MS showing its unique feature for rapid sample analysis. In PSI-MS, single sample analysis can be completed within 3 min for sample preparation and analysis. In this work, it should note that paper sampling can be completed within 1 min, while PSI-MS analysis can also be completed within 1 min, showing a rapid sampling and analysis processes. These data demonstrated that paper strips can be used for adsorptive sampling and direct MS analysis of chemicals from a burning cigarette.

**TABLE 1:** Analytical performances of nicotine detection in complex matrices with different methods.

Methods	Matrices	Precision	Linearity ranges	RSD	Analytical time	Limit of detection		References
						LOD	LOQ	
GC-MS	Tobacco products	101–102 %	0.01–8.18 mg/g	< 2.0 %	> 30 min	0.06 mg/g	NA	Stanfill et al. (2009)
GC-MS	Biofluid	94.5–101.2%	1–5000 ng/mL	< 1.1 %	> 30 min	0.25	0.8 ng/ml	Massadeh et al. (2009)
LC-MS	Biofluid	85.7–98.2 %	1–5000 ng/mL	< 7.5 %	> 30 min	0.26 ng/ml	0.9 ng/ml	Massadeh et al. (2009)
LC-MS	Biofluid	95–116 %	2–100 ng/mL	< 5.0 %	> 30 min	NA	1.8 ng/ml	Byrd et al. (2005)
LC-MS	Biomaterials	107–122%	0.01–0.04 mg/kg	< 10 %	> 30 min	1.0 μg/kg	2.0 μg/kg	Cavalieri et al. (2010)
PSI-MS	Tobacco smoke	76–124 %	1.0–500 ng/mL	13.5 %	< 5 min	0.2 ng/ml	1.0 ng/ml	This Work

### Differentiating tobacco smoke from different cigarettes

To investigate mass spectral fingerprints and specificity of tobacco smoke from different cigarettes, PSI-MS-based precise analysis coupled with multivariate variable analysis of tobacco smoke was performed to differentiate tobacco smoke from different cigarettes in this work. Monitoring markers of tobacco smoke would be useful for cigarette management and production. In this work, three types (type A, type B, and type C) of commercial cigarettes were compared; their characteristic spectral data under positive were analyzed.

**FIGURE 4:**
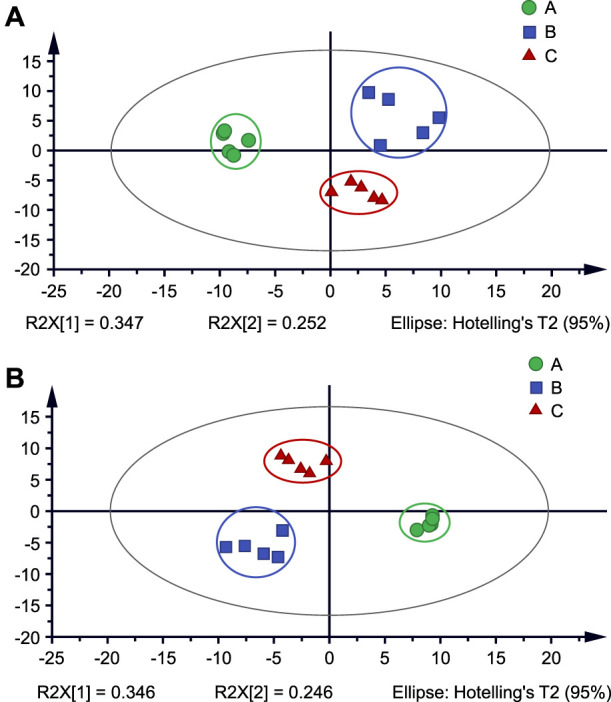
**(A)** PCA analysis and **(B)** PLS-DA analysis of tobacco smoke from three types (A–C) of cigarettes.

To better understand the differences between cigarettes, PCA plots of three types of cigarettes were obtained based on their MS data to compare their chemical characteristics, as shown in Figure 4A. These data clearly show that clusters of three types were well separated in PCA plots. This result suggests that although most the cigarettes have a lot of the same chemicals as the main ingredients of tobacco smoke, their contents and concentrations could be significant difference under different processing techniques and flavor substances. PLS-DA plots were further generated with variable importance in the projection (VIP) scores to seek the special markers. As shown in Figure 4B, these clusters clearly show that three types of cigarettes were well separated in PLS-DA plots. According to the VIP values (cutoff > 1.0), various ions were proposed to be marker candidates (Supplementary Table S1). Moreover, these ions were matched to PubChem databased to search possible chemicals. As listed in Supplementary Table S1, proposed chemicals were listed with a credible error estimation (Delta <5 ppm). These results show that PSI-MS could be a potential tool for investigating the chemical profiling of tobacco smoke.

## Conclusion

In conclusion, we applied an ambient MS method with PSI technique and paper-based auto-smoking sampling method to explore chemical profiling of different tobacco smoke. The advantage of paper sampling coupled with PSI-MS is that paper strip can be used for direct adsorptive sampling of tobacco smoke from burning cigarettes without any sample preparation and chromatography separation, and collected smoke samples were then directly analyzed by PSI-MS by adding a drop of methanol. Direct sampling and analysis of each sample can be completed within minutes. In this work, nicotine, the typical maker of tobacco smoke samples, was successfully detected by PSI-MS. The sampling conditions were optimized. Differentiating different cigarettes was also demonstrated by using PCA and PLS-DA analysis. Overall, our results show that PSI-MS could be a simple, rapid, and effective analytical tool for investigating the chemical properties of tobacco smoke.

## Data Availability

The original contributions presented in the study are included in the article/Supplementary Material; further inquiries can be directed to the corresponding author.
