# Supplementary material for: Direct adsorption sampling and ambient mass spectrometry analysis of tobacco smoke with porous paper strips
Source: Front Chem. 2022 Oct 26;10:1037542. doi: 10.3389/fchem.2022.1037542 (PMC9643588; doi:10.3389/fchem.2022.1037542)
Supplement: Supplementary file 1 [file Table1.DOCX]

**Table S1.** Ions of special makers (VIP > 1.0) and proposed chemicals (error < 5 ppm) of tobacco smoke.

| Measured ions (m/z) | VIP value | Theoretical value (m/z) | Formula | Adduct | Delta (ppm) |
| --- | --- | --- | --- | --- | --- |
| 106.0649 | 1.35678 | 106.0651 | C7H7N | M+H | 2 |
| 195.1856 | 1.33227 | 195.1856 | C12H22N2 | M+H | 0 |
| 120.0807 | 1.3217 | 120.0808 | C8H9N | M+H | 1 |
| 133.0765 | 1.30468 | 133.0760 | C6H12O3 | M+H | 4 |
| 117.0546 | 1.30052 | 117.0546 | C5H8O3 | M+H | 0 |
| 257.0448 | 1.29185 | 257.0444 | C14H8O5 | M+H | 1 |
| 309.2033 | 1.28333 | 309.2036 | C16H30O4 | M+Na | 1 |
| 130.0610 | 1.28128 | 130.0611 | C4H7N3O2 | M+H | 1 |
| 124.0756 | 1.26559 | 124.0757 | C7H9NO | M+H | 2 |
| 231.1305 | 1.25628 | 230.1307 | C15H18O2 | M+H | 2 |
| 148.0730 | 1.22664 | 148.0733 | C7H11NO | M+Na | 2 |
| 132.0806 | 1.22555 | 132.0808 | C9H9N | M+H | 1 |
| 191.1120 | 1.2209 | 168.1123 | C6H12N6 | M+Na | 1 |
| 193.0498 | 1.21597 | 193.0495 | C10H8O4 | M+H | 1 |
| 152.1075 | 1.21282 | 152.1070 | C9H13NO | M+H | 3 |
| 177.0505 | 1.20428 | 177.0506 | C5H8N2O5 | M+H | 1 |
| 129.0485 | 1.20424 | 129.0481 | C5H8N2S | M+H | 1 |
| 186.1123 | 1.19729 | 186.1125 | C9H15NO3 | M+H | 3 |
| 460.1525 | 1.19715 | 460.1526 | C21H23N7O2S | M+Na | 0 |
| 217.1300 | 1.19635 | 217.1295 | C8H16N4O3 | M+H | 2 |
| 186.0965 | 1.19625 | 186.0961 | C4H13N5O2 | M+Na | 2 |
| 185.0975 | 1.19358 | 185.0971 | C8H18OS | M+Na | 2 |
| 258.1598 | 1.1921 | 258.1596 | C15H23O2 | M+Na | 1 |
| 237.1822 | 1.19155 | 237.1825 | C13H26O2 | M+Na | 1 |
| 151.0395 | 1.18949 | 151.0390 | C8H6O3 | M+H | 4 |
| 131.0701 | 1.18701 | 131.0703 | C6H10O3 | M+H | 1 |
| 236.1770 | 1.18492 | 236.1776 | C15H23O2 | M+H | 3 |
| 347.2192 | 1.18102 | 347.2193 | C19H32O4 | M+Na | 1 |
| 181.1457 | 1.18077 | 181.1461 | C11H18NO | M+H | 2 |
| 169.0858 | 1.17516 | 169.0859 | C9H12O3 | M+H | 1 |
| 138.0890 | 1.17228 | 138.0895 | C6H14NO | M+Na | 0 |
| 236.1118 | 1.17159 | 236.1118 | C8H15N5O2 | M+Na | 0 |
| 150.0900 | 1.16853 | 150.0889 | C7H13NO | M+Na | 1 |
| 167.1432 | 1.15777 | 167.1430 | C11H18O | M+H | 3 |
| 149.0205 | 1.15414 | 149.0209 | C6H6O3 | M+Na | 4 |
| 153.1368 | 1.15196 | 153.1362 | C7H18N2 | M+Na | 1 |
| 146.0602 | 1.15062 | 146.0600 | C9H7NO | M+H | 0 |
| 193.1701 | 1.15032 | 193.1699 | C12H20N2 | M+H | 1 |
| 175.0710 | 1.14953 | 175.0713 | C6H10N2O4 | M+H | 1 |
| **Table S1. Continued.** | | | | | |
| Measured ions (m/z) | VIP value | Theoretical value (m/z) | Formula | Adduct | Delta (ppm) |
| 163.0712 | 1.14688 | 163.0713 | C5H10N2O4 | M+H | 1 |
| 159.0668 | 1.14004 | 159.0665 | C8H6N4 | M+H | 2 |
| 314.1218 | 1.13942 | 314.1219 | C13H25NO2S2 | M+Na | 0 |
| 123.0401 | 1.13294 | 123.0400 | C2H6N2O4 | M+H | 1 |
| 149.0601 | 1.12444 | 149.0597 | C9H8O2 | M+H | 3 |
| 167.0334 | 1.11506 | 167.0315 | C6H8O4 | M+Na | 3 |
| 162.9101 | 1.11416 | 162.9107 | C2H4S3 | M+K | 3 |
| 242.0695 | 1.11245 | 242.0690 | C11H13N3O | M+K | 2 |
| 153.0514 | 1.1098 | 153.0515 | C4H12N2S2 | M+H | 0 |
| 164.1070 | 1.10581 | 164.1070 | C10H13NO | M+H | 0 |
| 169.0794 | 1.10541 | 169.0794 | C8H12N2S | M+H | 0 |
| 241.0700 | 1.10112 | 241.0696 | C10H10N4O2 | M+Na | 2 |
| 139.0388 | 1.09912 | 139.0390 | C7H6O3 | M+H | 1 |
| 243.0710 | 1.09759 | 243.0719 | C8H18O4S2 | M+H | 4 |
| 217.0495 | 1.08398 | 217.0495 | C12H8O4 | M+H | 0 |
| 161.0601 | 1.07623 | 161.0597 | C10H8O2 | M+H | 2 |
| 164.1040 | 1.06049 | 164.1046 | C8H15NO | M+Na | 4 |
| 135.0405 | 1.05967 | 135.040 | C3H6N2O4 | M+H | 3 |
| 163.1232 | 1.05076 | 163.1230 | C10H14N2 | M+H | 1 |
| 164.1145 | 1.04654 | 164.1142 | C4H13N5O2 | M+H | 2 |
| 329.2452 | 1.0437 | 329.2451 | C20H34O2 | M+Na | 2 |
| 165.1270 | 1.04272 | 165.1274 | C11H16O | M+H | 2 |
| 327.2300 | 1.04233 | 327.2294 | C20H32O2 | M+Na | 2 |
| 347.0900 | 1.04057 | 347.0891 | C16H20O6 | M+K | 2 |
| 139.1205 | 1.04026 | 139.1206 | C6H16N2 | M+Na | 0 |
| 213.1169 | 1.03828 | 213.1168 | C9H16N4S | M+H | 0 |
| 136.0731 | 1.03767 | 136.0733 | C6H11NO | M+Na | 1 |
| 173.0806 | 1.03253 | 173.0808 | C8H12O4 | M+H | 1 |
| 157.0775 | 1.03143 | 157.0770 | C5H14N2S | M+Na | 3 |
| 459.1500 | 1.02991 | 459.1497 | C20H26O12 | M+H | 1 |
| 169.0500 | 1.02129 | 169.0500 | C6H12NO2 | M+K | 0 |
| 163.3467 | 1.02129 | Unknow | Unknow | Unknow | Unknow |
| 183.1983 | 1.01472 | Unknow | Unknow | Unknow | Unknow |
| 185.1075 | 1.00929 | 185.1073 | C12H12N2 | M+H | 1 |
| 203.0523 | 1.00713 | 203.0526 | C6H12O6 | M+Na | 2 |
